# Key Considerations for Designing Clinical Studies to Evaluate Digital Health Solutions

**DOI:** 10.2196/54518

**Published:** 2024-06-17

**Authors:** Elaina Bolinger, Benoit Tyl

**Affiliations:** 1 Integrated Evidence Generation & Business Innovation Bayer AG Berlin Germany; 2 Integrated Evidence Generation & Business Innovation Bayer HealthCare SAS La Garenne Colombes France

**Keywords:** evidence generation, clinical robustness, clinical trials, digital health, solutions, digital health solutions, DHS, health care system, patients, patient, individuals, individual, healthcare system, control arm adaptations, randomization methods, real-world data, platform research

## Abstract

Evidence of clinical impact is critical to unlock the potential of digital health solutions (DHSs), yet many solutions are failing to deliver positive clinical results. We argue in this viewpoint that this failure is linked to current approaches to DHS evaluation design, which neglect numerous key characteristics (KCs) requiring specific scientific and design considerations. We first delineate the KCs of DHSs: (1) they are implemented at health care system and patient levels; (2) they are “complex” interventions; (3) they can drive multiple clinical outcomes indirectly through a multitude of smaller clinical benefits; (4) their mechanism of action can vary between individuals and change over time based on patient needs; and (5) they develop through short, iterative cycles—optimally within a real-world use context. Following our objective to drive better alignment between clinical evaluation design and the unique traits of DHSs, we then provide methodological suggestions that better address these KCs, including tips on mechanism-of-action mapping, alternative randomization methods, control-arm adaptations, and novel end-point selection, as well as innovative methods utilizing real-world data and platform research.

## Introduction

Digital health solutions (DHSs) can improve health care access, patient equity, operational efficiency, and cost-effectiveness for health care organizations while delivering clinical outcomes for patients. Despite the rapid proliferation of DHSs, most lack convincing evidence supporting their clinical impact [1], preventing their uptake within the health care system and blocking subsequent development cycles required to realize their full clinical potential. Closing this evidence gap and producing strong evidence in a timely and cost-effective manner is critical for the establishment of trust in DHSs and imperative for their adoption and implementation—prerequisites for unlocking digital health’s potential to truly transform health care [2].

Randomized controlled trials (RCTs) using placebo control groups; individual randomization; and strict, arguably artificial clinical settings are the gold standard for evaluating drug efficacy and safety [3]. But contrary to pharmacological treatments, in which efficacy is driven by the modulation of biology at the molecular level, DHSs belong to a specific interventional class known as “complex interventions” [4]. By definition, the impact of this type of intervention is not solely biological but also encompasses psychological, behavioral, and systems-level effects, indicating context-dependency [5]. These attributes may, for example, help explain why effect sizes for pharmaceutical products are generally higher in traditional RCTs compared to research conducted under conditions reflecting real-world conditions [6,7], whereas DHS effect sizes tend to be higher when using designs that mimic real-world conditions [8]. The unique attributes of DHSs and the “complex” class of interventions they deliver may therefore necessitate a more tailored approach to their evaluation to measure their true impact.

In this viewpoint, we discuss our perspective on what must be considered when designing clinical evaluations of DHSs. We specifically aimed to (1) delineate a series of key characteristics (KCs) of DHSs that make them different from pharmacological agents and should be considered while designing their evaluation, and (2) offer methodological solutions to adapt research to the requirements of DHSs, addressing randomization; control-arm design; end-point selection; and nontraditional, innovative adaptations.

## DHS Key Characteristics

### KC1: DHSs Are Often Implemented at Health Care System and Patient Levels

While patients may generate most of the data, DHSs frequently involve other individuals such as health care professionals (HCPs) and caregivers, who must integrate the solutions into their workflows for the DHS to reach full effectiveness. The benefits of using DHSs frequently expand beyond patients to caregivers, HCPs, and the whole health care organization. For instance, implementing a remote patient monitoring DHS in a single health care center can have permeating systemic effects, with the product improving outcomes of the patients monitored as well as those of the other patients, either by decreasing readmission and then shifting clinical staff resources to preventive care or by improving health care efficiency (streamlined workflow, optimized patient engagement strategies, etc). Within an evaluation setting, HCPs in the control group may adapt their behavior via learning from those included in the intervention group and change their clinical strategies (ie, leading to contamination). Therefore, it is important to control not just at the patient level but also at the health care site level [9].

### KC2: DHSs Are “Complex” Interventions

Owing to their multiuser designs and dependencies on real-world health care systems, the mechanism of action (MoA) of DHSs depends on the historical, situational, environmental, and psychological factors in which treatments are delivered. Contextual dependencies include but are not limited to: HCP characteristics; patient-HCP relationships; perceived intervention credibility; delivery modality; psychological state of the individual; and societal, economic, and cultural factors. Even seemingly small nuances, such as a patient having previously been asked about their health, can have a significant impact on their health-related behavior and consequently also on health outcomes [10-12]. Many of these contextual factors are unintentionally, artificially modified in pharmaceutical RCTs to maximize internal validity. For instance, the Hawthorn effect, which is an increase in engagement when people are observed [13], and other research participation effects [14] are controlled between groups by using placebos. Yet this engagement is in fact a designed benefit of several DHSs; therefore, adding measurement points that would not occur in real-world situations to a DHS control group can render the control noninert. For example, measuring and regularly reporting blood pressure improves engagement and hypertension control [15], and similarly, reporting symptoms improves cancer self-management practices [16]. The artificial contexts and measurement conditions traditionally used in pharmaceutical RCTs can therefore limit the ecological validity of how the DHS is used. Consequently, conclusions drawn from such designs may lack external validity.

### KC3: DHSs May Drive Multiple Clinical Outcomes Indirectly Through a Multitude of Smaller Clinical Benefits

DHSs are frequently “holistic” interventions that provide a broad spectrum of solutions to induce diverse changes, mainly behavioral, which then work as levers to deliver clinically meaningful outcomes. For instance, a remote patient monitoring platform for patients with diabetes that reduces time to treatment adjustment and thereby improves pharmaceutical adherence should consequently improve blood glucose regulation. Similarly, any improvement of sleep behavior by a sleep coaching DHS may also improve cardiovascular risk through better exercise, decreased weight, better eating habits, decreased cholesterol, and improved glucose regulation. While the magnitude of effect of the DHS on each of these end points may be limited, the cumulative effects may lead to a clinically significant benefit. Choosing a single primary clinical end point during evaluation therefore may not capture the complexity of the DHS’s intended purpose nor its true performance.

### KC4: The MoA Driving Clinical Outcomes in DHSs Can Vary Between Individuals and Change Over Time Based on Patient Needs

Many DHS interventions include several separate, situationally activated components or features (HCP-facing interface, symptom monitoring, activity tracking, etc) that can bring unique benefits. For example, one patient might use feature A, which indirectly improves cholesterol levels; another might prefer feature B and experience an improvement in blood pressure, with smaller changes observed in cholesterol; and some patients might benefit from both. The effect size of the intervention for a single end point may therefore depend on how the product is used. Moreover, the second patient may begin to prefer feature A, but only later during use. The effect size of the intervention for a single end point can therefore be weakened when the DHS as a whole rather than specific feature use is considered as the intervention. What is more, many DHSs employ adaptive algorithms that are specifically designed to adjust the intervention over time. Depending on the DHS being considered, researchers might therefore anticipate that due to user-dependent variability in the MoA and emerging synergistic influences (ie, better product engagement following use-based personalization), the clinical impact may require a longer treatment period to be detected.

### KC5: DHSs Develop Through Short, Iterative Cycles—Often Within a Real-World Use Context

Contrary to active pharmacological ingredients that cannot change, DHSs continuously evolve in terms of their functionality with each subsequent release. Because DHSs must meet their users’ needs in the real world, an environment where many factors remain unknown [17], developers often perform accelerated, iterative tests to both discover product performance factors and adapt them before the next release. Due to the number of factors that must be discovered, how they can influence each other, and how influential certain factors can be (ie, a seemingly small adjustment of written content to an audio format might completely change engagement and therefore clinical outcomes), the solution often must stay moderately malleable, as product design hypotheses are validated over a continuum of nonclinical tests. Clinical researchers often opt to evaluate a “frozen” version of the product to optimize homogeneity in the intervention arm, but these approaches may limit external validity, as the marketed solution may drastically evolve over time and the versions tested by the RCT may be outdated at the time of study completion.

## Recalibrating Research Design to Meet the Scientific Canvas of DHSs

**Figure 1:**
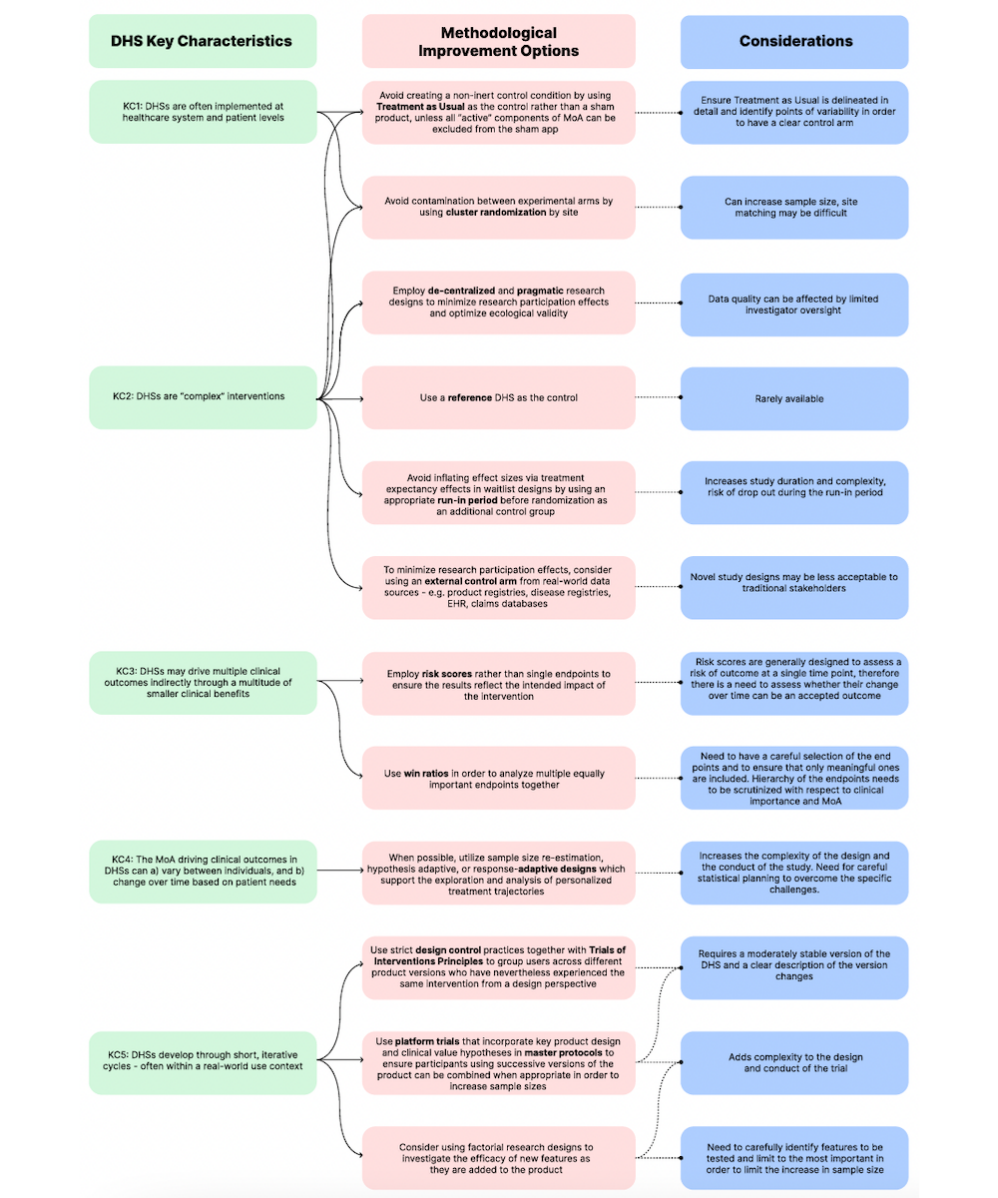
Overview of key characteristics (KCs) of the digital health solutions (DHSs) and potential methodological solutions to improve their clinical assessment. EHR: electronic health record; MoA: mechanism of action. For a higher-resolution version of this figure, see Multimedia Appendix 1.

Considering the KCs described above, alternative assessment strategies are needed to address the unique challenges of DHS evaluation while maintaining internal validity (unbiased study design), external validity (applicability to different contexts), and ecological validity (generalizability to real-world settings). The solutions discussed below are summarized in Figure 1.

The first step to design a DHS evaluation is to take the time to delineate the MoA of the solution and the context. This includes a map of behavioral changes required from patients and all other individuals who are affected by the product (KC1), as well as the contextual environment of use (KC2). Given the limitations of an active control arm described above (KC2), the use of usual care—usually named treatment as usual (TAU)—may serve as an appropriate control. TAU corresponds to the actual routine care and may differ from the state-of-the-art, guideline-adherent clinical care. Therefore, a prerequisite for this approach would be a detailed understanding of the procedural standard of care, which includes, but is not limited to, *local* (ie, site-specific) procedures, treatment recommendations, information delivered (eg, health literacy counseling), and frequency of contacts with HCPs. This task is nontrivial, as evidenced by standard-of-care descriptions being generally underreported [18], and can pose exceptional challenges due to the number of factors in a real-world environment. Combining patient journeys, DHS user journeys, and HCP patient management protocols is a helpful first step to identify how the product induces change within its context of use. Process evaluation techniques [19] can also be employed to extract MoAs for complex interventions during preliminary implementation research [5]. With the MoA delineated in detail, one can begin designing an appropriate evaluation employing a fit-for-purpose scientific approach.

As many have suggested before [9,20], cluster randomization by health care site, cities, or region is a methodologically sound approach to control for contamination between arms while taking into account DHS benefits, which depend on factors extending beyond the treated patient (KC1). This design, however, can necessitate larger sample sizes, and site matching can be challenging. Pragmatic designs [9], which anchor RCTs in real-world settings and minimize research-related contact outside of TAU, can help maximize the ecological validity of both the experimental (+TAU) and TAU arms (KC2). Keeping the trial decentralized can minimize research-related contact while increasing trial accessibility and make the research population more representative of the target population (KC2). While DHSs, which digitally collect data by nature, are particularly suitable for such designs, limitations secondary to the reduced investigators oversight, including data quality and outcome assessment, persist.

Even with these tools in mind, designing a control arm with high internal validity remains challenging when evaluating DHSs. A reference DHS is an appropriate control but rarely exists. While some have argued that a “sham” app (ie, as defined by the Food and Drug Administration, an app without the “active” features anticipated to drive the MoA [21]) offers optimal internal validity, in that it is a form of “placebo” technology, this design can pose critical threats to external and ecological validity, particularly when the MoA itself is not fully understood. Providing even minimalistic digital support that is nevertheless seemingly endorsed by HCPs (as would be the case in a clinical trial) might still produce a similar, albeit muted, effect as the DHS. For example, a sham app delivering some health literature is not inert, as it can improve health literacy and consequently health-related behaviors. The use of a sham app as a control is therefore challenging unless it is possible to exclude all active ingredients of the MoA, including engagement and interaction with the real world, from the sham app. On the other hand, the use of TAU as a control can potentially inflate effect size with some interventions (KC2). The traditional “waitlist” design used in medical device evaluation may also be appropriate but can inflate effect sizes by decreasing behavioral activation in the control arm (in essence, inducing the opposite of the Hawthorne effect) [22,23]. Researchers considering having a waitlist TAU design as the comparator might use an appropriate *run-in period* before randomization, during which both study arms receive TAU to minimize the influence of treatment expectancy effects [24] within the comparator arm (KC2). Finally, it is important to consider how the nature of the intervention and the control arm may impact the risk of attrition in the study. Beyond minimizing attrition in general, it is crucial to ensure that the attrition rates between the active and control arms are balanced. Significant differences in dropout rates between both arms may lead to variations in the clinical characteristics of the remaining patients, potentially biasing the overall analysis.

DHS researchers may also explore alternate evaluation designs. The use of synthetic control arms built using real-world data sources such as electronic health records, claims databases, and disease registries may address certain limitations inherent to the conventional control group. However, the creation of these synthetic arms needs to be considered carefully, as they pose their own set of challenges and are not devoid of their own limitations. In case of products already on the market, epidemiological methods could be used to examine causal inference in product registry data—again minimizing research participation effects. These approaches may in some cases both be more cost-effective and provide higher quality data on product performance.

Designing a trial that accounts for the holistic impact of DHSs is also critical for demonstrating performance and efficacy. Contrary to pharmacological agents that usually improve markedly a single biomarker (hemoglobin A_1c_, C-reactive protein, etc), DHSs frequently improve modestly several biomarkers (physical activity, lipids, weight, etc; KC3). Here, rather than examining the effect on a single end point, researchers might employ a risk score that better reflects the holistic nature of the intervention. Win ratios are also an elegant way to analyze multiple, equally valuable end points [25]. Adaptive designs, such as those incorporating sample size re-estimation, hypothesis-adaptive, or response-adaptive designs [26], could also be both powerful and appropriate given the novel nature of many DHS interventions and the different use patterns and emergent interventions that can arise (KC4). Given the dynamic and personalized nature of DHSs, the evaluation design should also carefully consider anticipated effect size at each time point during planning.

**Figure 2:**
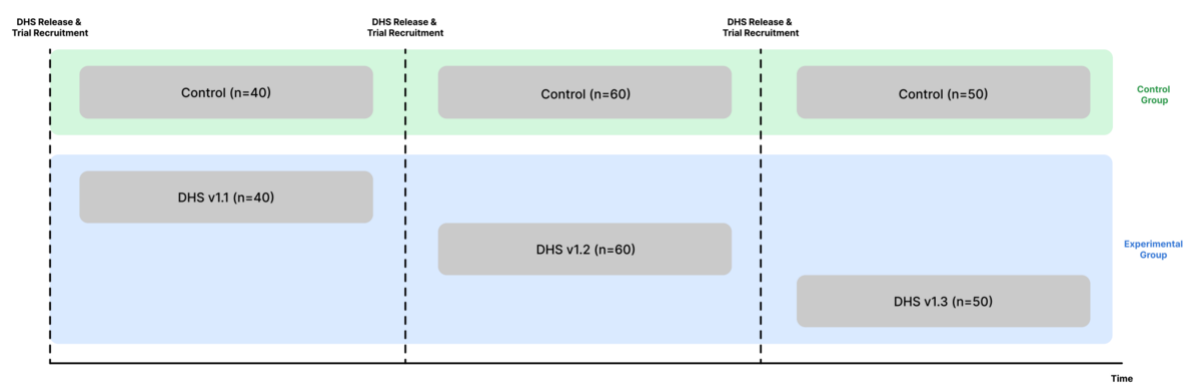
Example of design of a platform trial including 300 patients (150 per arm) enabling the evaluation of multiple versions of a DHS within one study. The figure illustrates the recruitment flow of the patients in the trial. Multiple versions of a DHS can be included in a single study when guidelines for identifying significant changes to study procedures and DHS solutions are delineated in advance. A matched control arm with similar recruitment procedures ensures comparability in the control group. DHS: digital health solution.

The above can greatly improve the evaluation of DHS clinical performance but do not address the practical issue of evaluating rapidly and continuously evolving DHSs (KC5). While there is no definitive way to address this, a few options exist beyond freezing the product. During evaluation planning, planned future modifications of the product can be scrutinized to determine whether they constitute major changes to the MoA or intended use and would therefore require protocol modification during study execution. By combining strict product design control practices with a Trials of Intervention Principles approach [27], researchers can aggregate groups of users who may have used product versions with minor design variations not anticipated to change the MoA, allowing the product to continue to organically develop (KC5). Platform trials that incorporate key product design and clinical value hypotheses in master protocols can be leveraged to have the successive versions of the DHS tested and the results of the substudies combined to maximize sample sizes, rather than performing several insufficiently powered successive trials, while keeping analyses anchored in clearly documented starting hypotheses and protocols (Figure 2) [20,28]. Likewise, factorial research designs can be used to investigate the efficacy of new features as they are added to the product [29,30].

## Conclusion

DHSs need to be rigorously evaluated to fulfill the needs of the various stakeholders, including HCPs, patients, regulators, and payers. In this viewpoint, we proposed that their unique characteristics and MoA create a critical need for tailored, innovative approaches that move beyond traditional pharmaceutical RCTs in order to create fit-for-purpose, robust, and scientifically optimized evaluations. While no one-size-fits-all design exists, both researcher and stakeholders should embrace nontraditional methodologies that match the key characteristics of this emerging type of health care intervention.

## Appendix

Multimedia Appendix 1 Overview of key characteristics (KCs) of the digital health solutions (DHSs) and potential methodological solutions to improve their clinical assessment. EHR: electronic health record; MoA: mechanism of action.

## Abbreviations

DHS: digital health solution
HCP: health care professional
KC: key characteristic
MoA: mechanism of action
RCT: randomized controlled trial
TAU: treatment as usual
