# Supplementary figures and images for: Key Considerations for Designing Clinical Studies to Evaluate Digital Health Solutions
Source: J Med Internet Res. 2024 Jun 17;26:e54518. doi: 10.2196/54518 (PMC11217703; doi:10.2196/54518)

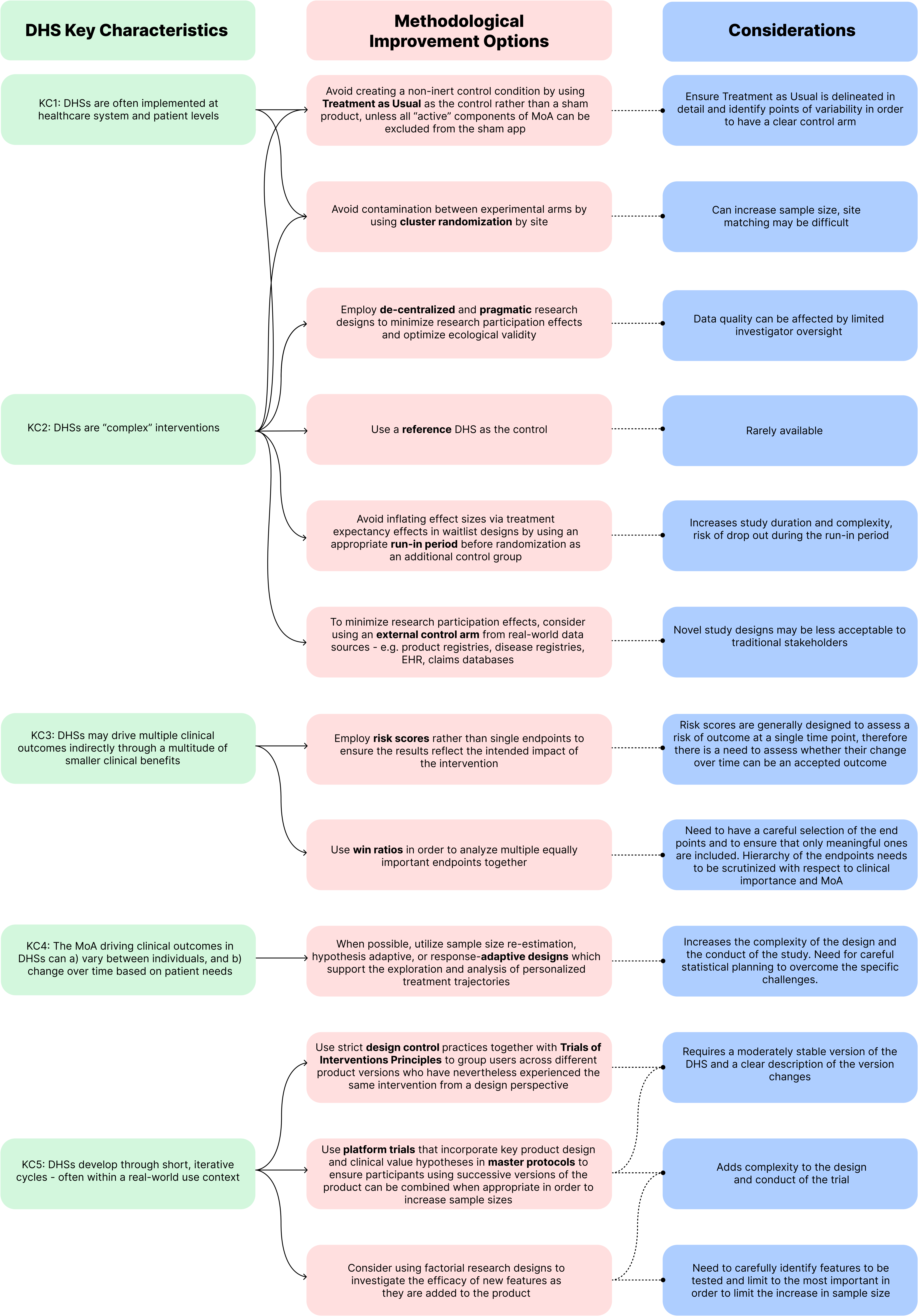

Supplement: Multimedia Appendix 1 [file jmir_v26i1e54518_app1.pdf]
